# Supplementary material for: A plant-specific clade of serine/arginine-rich proteins regulates RNA splicing homeostasis and thermotolerance in tomato
Source: Nucleic Acids Res. 2024 Aug 24;52(19):11466–80. doi: 10.1093/nar/gkae730 (PMC11514476; doi:10.1093/nar/gkae730)
Supplement: gkae730_Supplemental_Files [file gkae730_supplemental_files.zip › SUPPLEMENTARY DATA.pdf]

SUPPLEMENTARY DATA

Supplementary Tables

Supplementary Table S1. List of oligonucleotides

Supplementary Datasets

Supplementary Dataset S1. List of alternative splicing events in leaves of wild type plants under control and heat stress conditions.

Supplementary Dataset S2. List of differentially expressed genes in leaves of wild type plants under control and heat stress conditions.

Supplementary Dataset S3. Gene ontology term of DEG and DAS genes wild type plants under control and heat stress conditions.

Supplementary Dataset S4. List of alternative splicing events differentially regulated in in leaves of single and double *rs2z* mutants compared to wild type plants under control and heat stress conditions.

Supplementary Dataset S5. Differentially expressed genes in tomato leaves of single and double *rs2z* mutant lines compared to wild type under control and heat stress conditions.

Supplementary Dataset S6. Binding sites of RS2Z35 and RS2Z36 on RNA of heat-stressed tomato leaves.

Supplementary Dataset S7. List of tomato genes with RS2Z35 and RS2Z36 binding sites

Supplementary Dataset S8. List of nucleotide motifs identified by de novo motif search for RS2Z35 and RS2Z36 binding sites.

Supplementary Dataset S9. Binding sites of RS2Z35 and RS2Z36 on RNA of heat-stressed tomato leaves and occurrence of GGAG, CUCC, UUUU and AAAA motifs.

Supplementary Figures

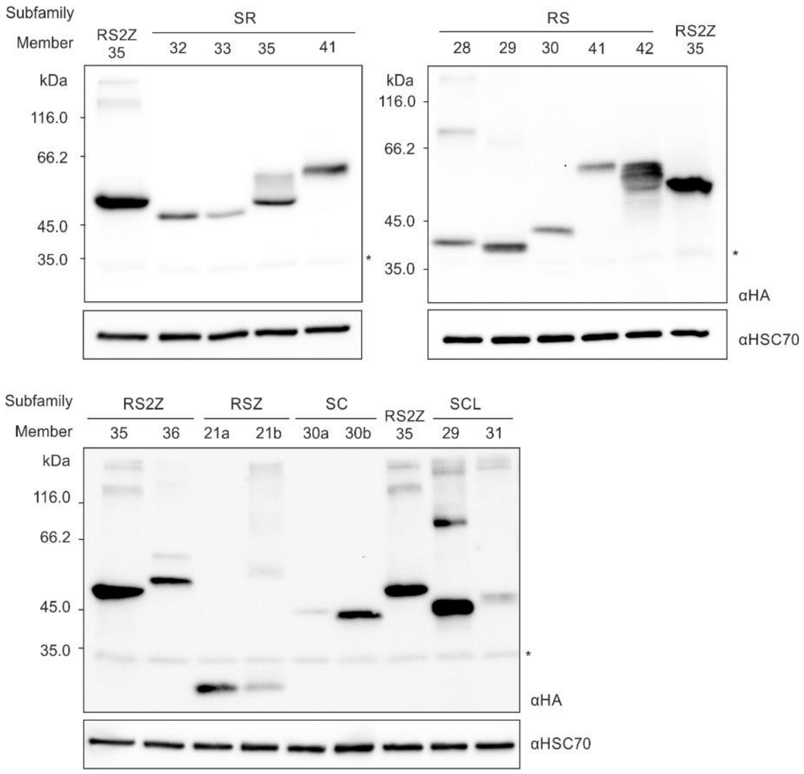

Supplementary Figure S1. Immunoblot analysis of HA-tagged SR proteins expressed in tomato mesophyll protoplasts. RS2Z35 is shown in all immunoblots for comparison. HSC70 is shown as loading control. The blots are Supplementary to Fig. 2c.

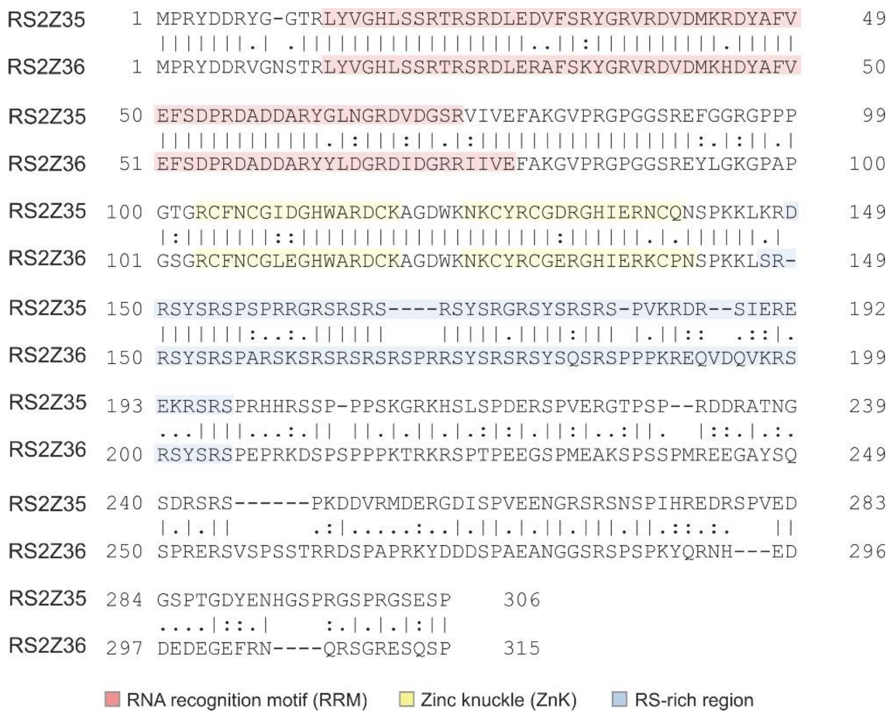

Supplementary Figure S2. Alignment of the protein sequence of RS2Z35 and RS2Z36. Red color indicates the RRM, yellow the ZnKs and blue the RS-rich region. This figure is Supplementary to Fig. 2d.

RS2Z35

WT:

MVFYPYDVPDYAGYPYDVPDYAGSYPYDVPDYAAQVPPRYDDRYGGTRLYVGHLSSRTRSRDLEDVFSRYGRVRDVD
MKRDYAFVEFSDPRDADDARYGLNGRDVDGSRVIVEFAKGVPRGPGGSREFGGRGPPPGTGRCFNCGIDGHWARDC
KAGDWKNKCYRCGDRGHIERNCQNSPKKLKRDRSYSRSPSPRRGRSRSRSRSYSRGRSYSRSRSPVKRDRSIEREEK
RSRSPRHHRSSPPPSKGRKHSLSPDERSPVERGTPSPRDDRATNGSDRSRSPKDDVRMDERGDISPVEENGRSRSNS
PIHREDRSPVEDGSPTGDYENHGSPRGSPRGSESP

ΔRRM:
MVFYPYDVPDYAGYPYDVPDYAGSYPYDVPDYAAQVPPRYDDRYGGTR~~LYVGHLSSRTRSRDLEDVFSRYGRVRDVD~~
~~MKRDYAFVEFSDPRDADDARYGLNGRDVDGSRVIVE~~FAKGVPRGPGGSREFGGRGPPPGTGRCFNCGIDGHWARDC
KAGDWKNKCYRCGDRGHIERNCQNSPKKLKRDRSYSRSPSPRRGRSRSRSRSYSRGRSYSRSRSPVKRDRSIEREEK
RSRSPRHHRSSPPPSKGRKHSLSPDERSPVERGTPSPRDDRATNGSDRSRSPKDDVRMDERGDISPVEENGRSRSNS
PIHREDRSPVEDGSPTGDYENHGSPRGSPRGSESP

ΔZnk:
MVFYPYDVPDYAGYPYDVPDYAGSYPYDVPDYAAQVPPRYDDRYGGTRLYVGHLSSRTRSRDLEDVFSRYGRVRDVD
MKRDYAFVEFSDPRDADDARYGLNGRDVDGSRVIVEFAKGVPRGPGGSREFGGRGPPPGT~~GRCFNCGIDGHWARDC~~
~~KAGDWKNKCYRCGDRGHIERNCQ~~NSPKKLKRDRSYSRSPSPRRGRSRSRSRSYSRGRSYSRSRSPVKRDRSIEREEK
RSRSPRHHRSSPPPSKGRKHSLSPDERSPVERGTPSPRDDRATNGSDRSRSPKDDVRMDERGDISPVEENGRSRSNS
PIHREDRSPVEDGSPTGDYENHGSPRGSPRGSESP

ΔRS:
MVFYPYDVPDYAGYPYDVPDYAGSYPYDVPDYAAQVPPRYDDRYGGTRLYVGHLSSRTRSRDLEDVFSRYGRVRDVD
MKRDYAFVEFSDPRDADDARYGLNGRDVDGSRVIVEFAKGVPRGPGGSREFGGRGPPPGTGRCFNCGIDGHWARDC
KAGDWKNKCYRCGDRGHIERNCQNSPKKLKR~~DRSYSRSPSPRRGRSRSRSRSYSRGRSYSRSRS~~PVKRDRSIEREEK
RSRSPRHHRSSPPPSKGRKHSLSPDERSPVERGTPSPRDDRATNGSDRSRSPKDDVRMDERGDISPVEENGRSRSNS
PIHREDRSPVEDGSPTGDYENHGSPRGSPRGSESP

RS2Z36

WT:
MVFYPYDVPDYAGYPYDVPDYAGSYPYDVPDYAAQVPPRYDDRVGNSTRLYVGHLSSRTRSRDLERAFSKYGRVRDV
DMKHDYAFVEFSDPRDADDARYYLDGRDIDGRRIIVEFAKGVPRGPGGSREYLGKGPAPGSGRCFNCGLEGHWARDC
KAGDWKNKCYRCGERGHIERKCPNSPKKLSRRSYSRSPARSKSRSRSRSRSPRRSYSRSRSYSQSRSPPPKREQVD
QVKRSRSYSRSPEPRKDSPSPPPKTRKRSPTPEEGSPMEAKSPSSPMREEGAYSQSPRERSVSPSSTRRDSPAPRKY
DDDSPAEANGGSRSPSPKYQRNHEDDEDEGEFRNQRSGRESQSP

ΔRRM:
MVFYPYDVPDYAGYPYDVPDYAGSYPYDVPDYAAQVP~~PRYDDRVGNSTRLYVGHLSSRTRSRDLERAFSKYGRVRDV~~
~~DMKHDYAFVEFSDPRDADDARYYLDGRDIDGRRIIVEFAK~~GVPRGPGGSREYLGKGPAPGSGRCFNCGLEGHWARDC
KAGDWKNKCYRCGERGHIERKCPNSPKKLSRRSYSRSPARSKSRSRSRSRSPRRSYSRSRSYSQSRSPPPKREQVD
QVKRSRSYSRSPEPRKDSPSPPPKTRKRSPTPEEGSPMEAKSPSSPMREEGAYSQSPRERSVSPSSTRRDSPAPRKY
DDDSPAEANGGSRSPSPKYQRNHEDDEDEGEFRNQRSGRESQSP

ΔZnk:
MVFYPYDVPDYAGYPYDVPDYAGSYPYDVPDYAAQVPPRYDDRVGNSTRLYVGHLSSRTRSRDLERAFSKYGRVRDV
DMKHDYAFVEFSDPRDADDARYYLDGRDIDGRRIIVEFAKGVPRGPGGSREYLGKGPAPGSG~~RCFNCGLEGHWARDC~~
~~KAGDWKNKCYRCGERGHIERKCPN~~SPKKLSRRSYSRSPARSKSRSRSRSRSPRRSYSRSRSYSQSRSPPPKREQVD
QVKRSRSYSRSPEPRKDSPSPPPKTRKRSPTPEEGSPMEAKSPSSPMREEGAYSQSPRERSVSPSSTRRDSPAPRKY
DDDSPAEANGGSRSPSPKYQRNHEDDEDEGEFRNQRSGRESQSP

ΔRS:
MVFYPYDVPDYAGYPYDVPDYAGSYPYDVPDYAAQVPPRYDDRVGNSTRLYVGHLSSRTRSRDLERAFSKYGRVRDV
DMKHDYAFVEFSDPRDADDARYYLDGRDIDGRRIIVEFAKGVPRGPGGSREYLGKGPAPGSGRCFNCGLEGHWARDC
KAGDWKNKCYRCGERGHIERKCPNSPKKL~~SRRSYSRSPARSKSRSRSRSRSPRRSYSRSRSYSQSRS~~PPPKREQVD
QVKRSRSYSRSPEPRKDSPSPPPKTRKRSPTPEEGSPMEAKSPSSPMREEGAYSQSPRERSVSPSSTRRDSPAPRKY
DDDSPAEANGGSRSPSPKYQRNHEDDEDEGEFRNQRSGRESQSP

RRM, Znk, RS domain (max length), RS domain (max percentage) 3xHA tag and linker sequence

Supplementary Figure S3. Sequences of deletion mutants. This figure is Supplementary to Fig. 2d. Red color indicates the RRM, yellow the ZnKs and blue the C-terminal with the RS-rich region shown underlined. The underlined N-terminal region indicated the 3xHA tag.

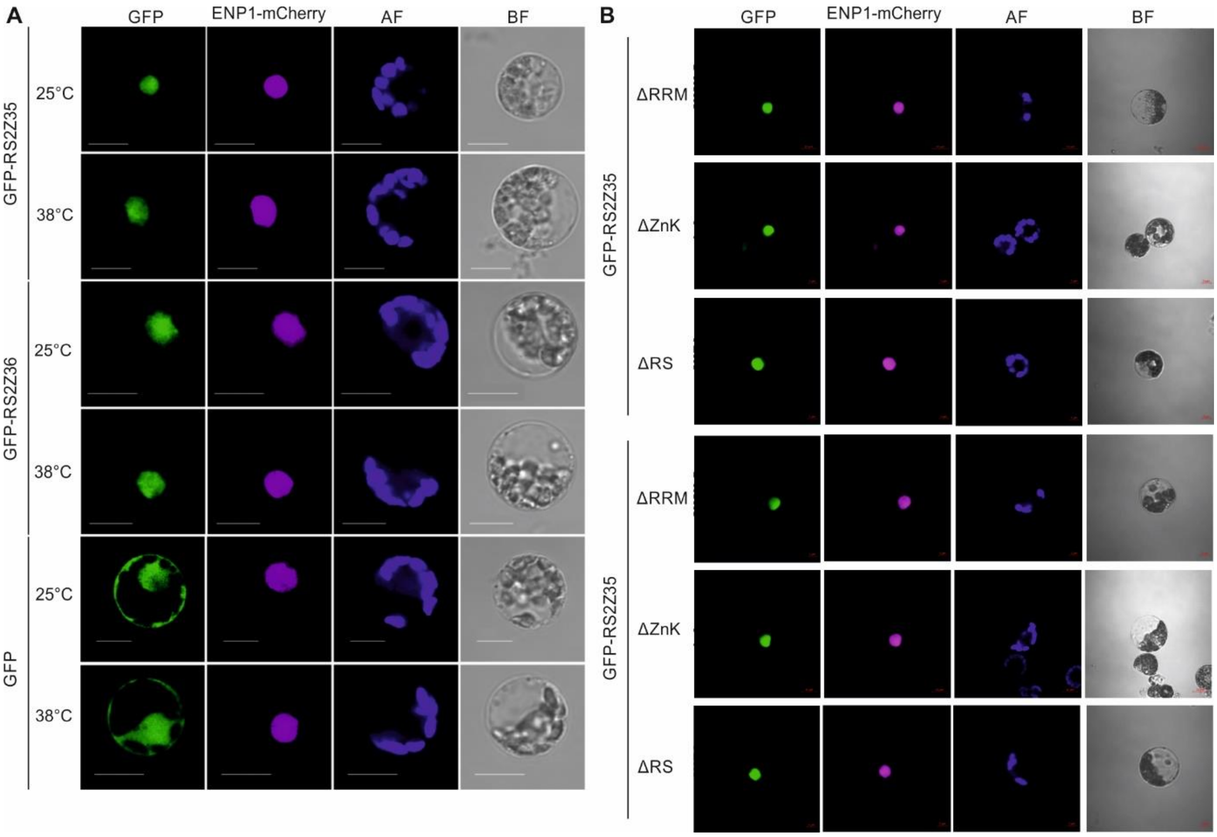

Supplementary Figure S4. Localization of GFP-tagged RS2Z35 and RS2Z36 domain deletion mutants in tomato protoplasts. Plasmids coding for the respective wild type (A) or mutant (B) RS2Z genes were co-transformed with plasmid expressing the nuclear marker ENP1-mCherry. A plasmid carrying an expression cassette of GFP alone was used as control (A). Protoplasts expressing GFP or the wild type proteins were either kept at 25°C or exposed for 1 hour to 38°C. Images were taken by a confocal laser scanning microscope. AF: autofluorescence; BF: Bright field.

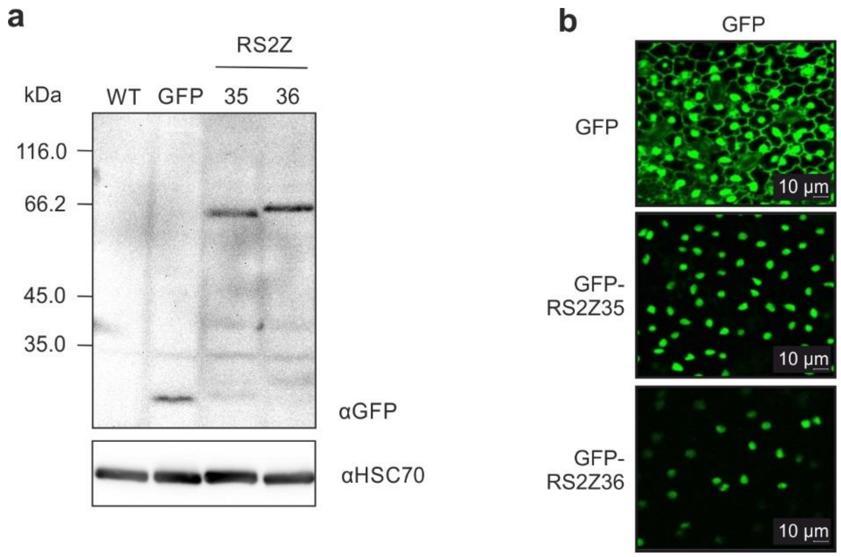

Supplementary Figure S5. Expression of GFP and GFP-RS2Z35, GFP-RS2Z36 constructs in leaves of transgenic tomato plants. (a) Immunoblot analysis of GFP and GFP-RS2Z proteins in the respective transgenic lines or WT plants. HSC70 is shown as loading control. (b) Localization of GFP or GFP-RS2Z proteins in cells of the adaxial site of leaves in the corresponding transgenic lines based on confocal laser scanning microscopy based on the GFP signal.

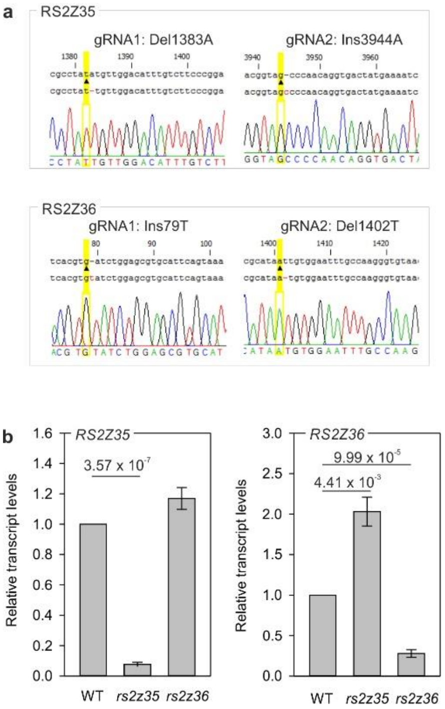

Supplementary Figure S6. CRISPR-Cas9 mutation of RS2Z genes. (a) Sequence of mutations in RS2Z35 and RS2Z36 genes. The position of the mutation is indicated in relation to the start nucleotide of the gene (based on ITAG4.0 annotation). The electropherograms showing the Sanger sequencing analysis was done in T3 generation in T-DNA free plants, carrying RS2Z homozygous mutations. (b) Relative transcript levels of *RS2Z35* and *RS2Z36* genes in the two RS2Z mutants compared to WT based on qRT-PCR. Error bars indicate standard deviation from 3 independent biological replicated. P-values are indicated on top, and correspond to pairwise t-test analysis. EF1a was used a reference gene for normalization. This is Supplementary to Figure 3.

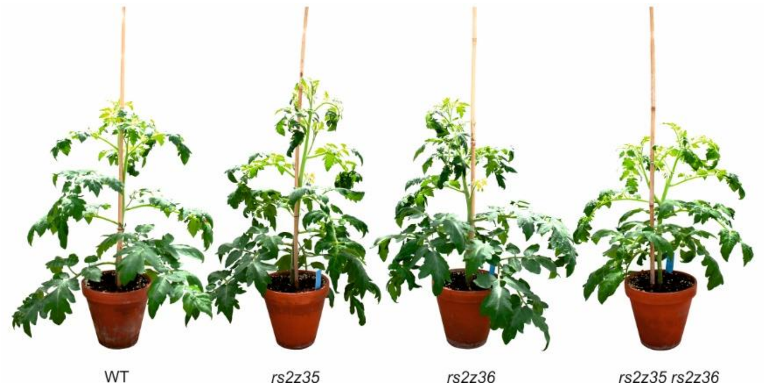

Supplementary Figure S7. Approximately 12-week old wild type, single and double *rs2z* mutants grown in the greenhouse (25°C / 16 h light (120 µS light intensity on the canopy) / 22°C / 8 h dark).

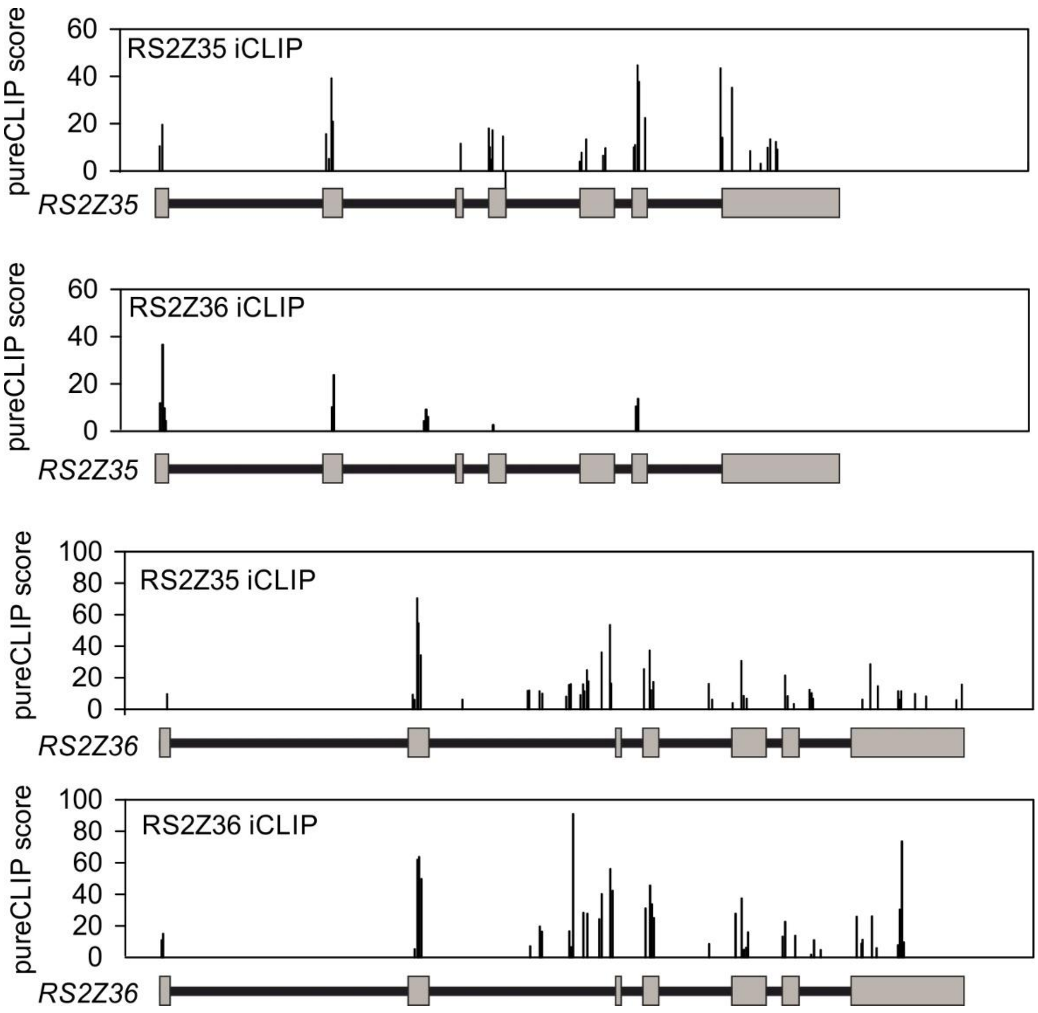

Supplementary Figure S8. Binding sites of RS2Z35 and RS2Z36 on the two genes. For each gene the exon/intron structure is indicated below. Y-scales show the PureCLIP score for each binding site.

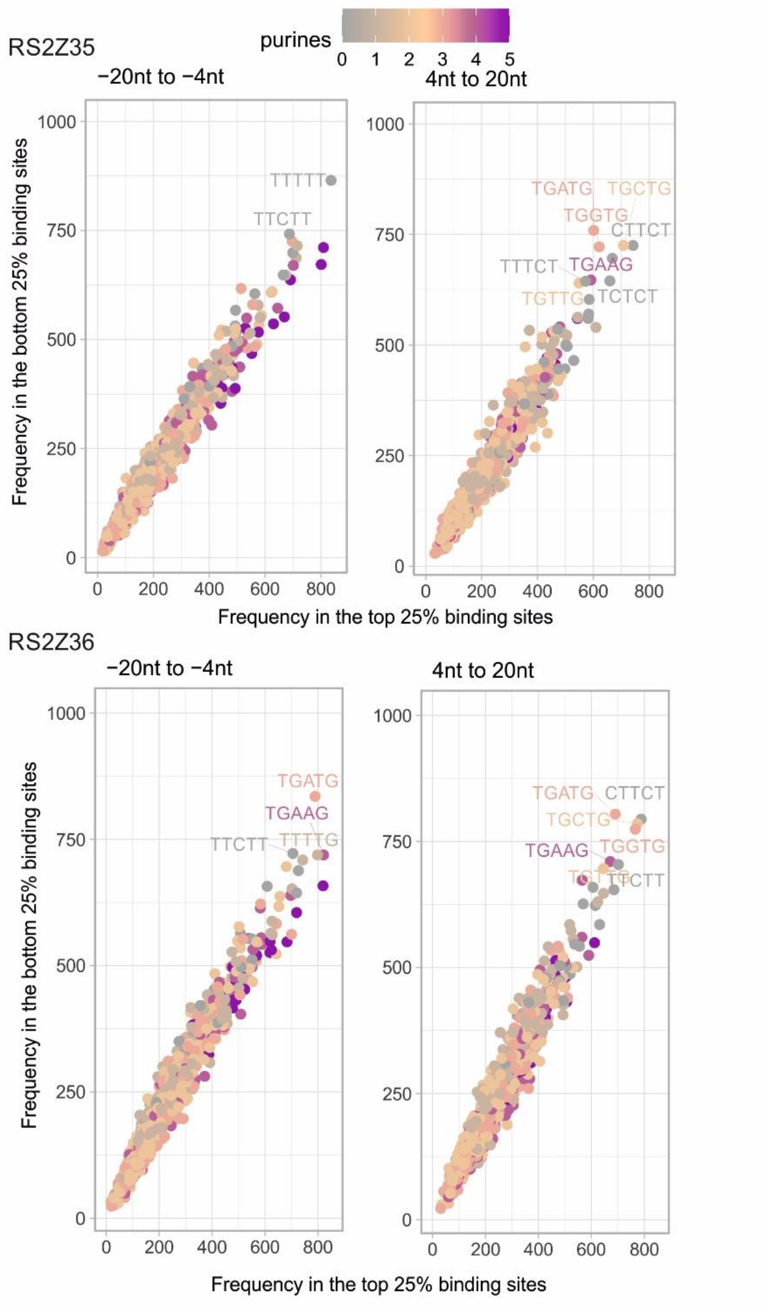

Supplementary Figure S9. Plots of all pentamers in a 17-nt window upstream (left, -20 nt to -4 nt) or downstream (right, 4 nt to 20 nt) of the top 25% and bottom 25% binding sites (based on the PureCLIP score as a proxy for binding site strength). The color of indicates the number of purines in the pentamers. This is Supplementary to Fig. 5i.

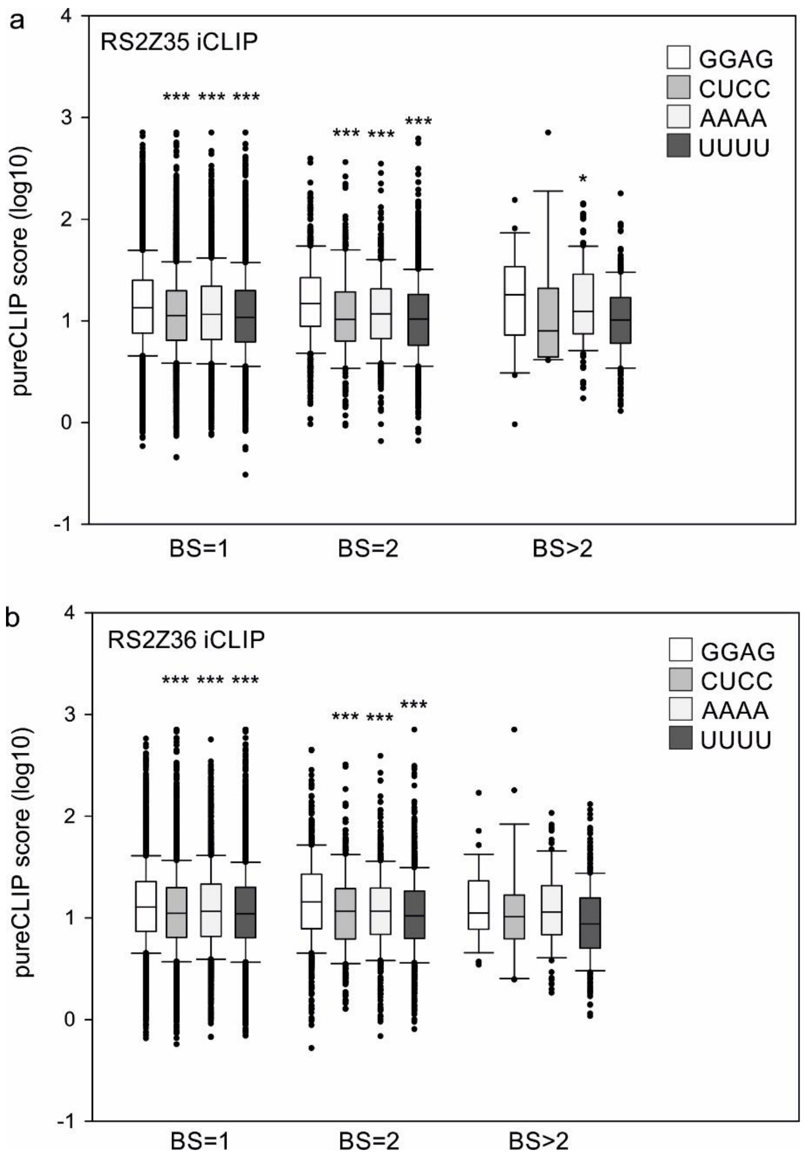

Supplementary Figure S10. Binding strength of RS2Z35 and RS2Z36 in presence of one, two or at least three GGAG motifs within a 15 nucleotide window. Binding strength is shown as pureCLIP score (log10). CUCC, AAAA and UUUU are shown as controls. Raw values can be found in Supplementary Dataset 9. Asterisks indicate statistically significant difference between one of control motifs and GGAG, based on ANOVA and Duncan's Multiple Range tests (* $p < 0.05$; *** $p < 0.001$).

Supplemental Table S1. List of oligonucleotides

**pRT-GFP-RS2Z**		
**Target**	**Forward (F)/Reverse (R)**	**Sequence (5' -> 3')**
NcoI-GFP-Acc65I amplification	F	TGGCCACCATGGGTAAAGGAG
	R	TCATGGTACCCGATCTAGTTCATC
**pRT-PHSP21.5-GFP-HSFA2-minigene**		
PHSP21.5 fragment	F	CTATGACCATGATTACGCCAGGTACCTTCATCCTAATTTTAC
	R	TATATGTTGTGTTGAGAATTCTCGAGACTATACACTGTAG
Linearisation of pRT-PCaMV35S::GFP-HSFA2-minigene	F	AATTCTCAACACAACATATACAAAAC
	R	GGCGTAATCATGGTCATAGC
**pICSL002208-RS2Z35- RNA1-2**		
sgRNA1-2	F	TGTGGTCTCAATTGGTGGCACACGCCTATATGTGTTTTAGAGCTAGAAATAGCAAG
	R	TGTGGTCTCAATTGTGGAAGACGGTAGCCCAACGTTTAGAGCTAGAAATAGCAAG
**pICSL002208-RS2Z36- RNA1-2**		
sgRNA1-2	F	TGTGGTCTCAATTGACGGACCCGTTCACGTGATCGTTTTAGAGCTAGAAATAGCAAG
	R	TGTGGTCTCAATTGTGATGGACGCCGCATAATTGGTTTTAGAGCTAGAAATAGCAAG
**pICH86966-P35S-GFP**		
GFP Fragment 1	F	CTAGGTCTCTGGAGTAGGCTTTACACTTTATGC
	R	TACGGTCTCTCTCTTTTCGTTGGGATCTTTC
GFP Fragment 2	F	TACGGTCTCTAGCGTACGGTCACAGCTTGTCTG
	R	ACAGGTCTCAAGAGGGACCACATGGTCCTT
**pICH86966-P35S-GFP-RS2Z**		
GFP-RS2Z fragment	F	CTAGGTCTCTGGAGTGAGTTAGCTCACTCATTAGG
	R	TACGGTCTCTAGCGTAGGGTTATTGTCTCATGAGC
**pRT-CaMV35S-HA-RS2Z deletion mutants**		

pRT-HA-RS2Z36ΔRRM	F	CGCTGCTCAGGTACCTGGTGTGCCACGTGGACCAGG CG
	R	TCCACGTGGCACACCAGGTACCTGAGCAGCGTAATC T GG
pRT-HA-RS2Z36ΔZnK	F	CCTGGGTCAGGTAGTCCTAAAAAACTCAGTAGACGC AG
	R	GTTTTTTAGGACTACCTGACCCAGGAGCGGGTCC
pRT-HA-RS2Z36ΔRS	F	CCTAAAAAACTCCCGCCACCAAAGAGAGAAC
	R	TGGTGGCGGGAGTTTTTTAGGACTATTTGGGCAT
pRT-HA-RS2Z35ΔRRM	F	GGTGGCACATTCGCCAAAGGGGTGCCTCGTG
	R	CTTTGGCGAATGTGCCACCATACCTGTC
pRT-35S-3HARS2Z35ΔZnk	F	GGTACTGGTAGCCCCAAGAAATTGAAACGTG
	R	TCTTGGGGCTGTCCTCCTCCAGGTACTGGT
Oligonucleotides used for cloning (continued)		
pRT-HA-RS2Z35ΔRS	F	GAAACGTGACCCTGTGAAGAGGGACCGTAG
	R	CTTCACAGG GTCACGTTTCAATTTCTTG
**CRISPR/Cas9 constructs**		
RS2Z35	sgRNA1	TGTGGTCTCAATTGGTGGCACACGCCTATATGTGTTT TAGAGCTAGAAATAGCAAG
	sgRNA2	TGTGGTCTCAATTGTGGAAGACGGTAGCCCAACGTT T TAGAGCTAGAAATAGCAAG
	tracrRNA sequence	TGTGGTCTCAAGCGACAAAAAAGCACCGACTCG
RS2Z36	sgRNA1	TGTGGTCTCAATTGACGGACCCGTTCACGTGATCGTT TTAGAGCTAGAAATAGCAAG
	sgRNA2	TGTGGTCTCAATTGTGATGGACGCCGCATAATTGGTT TTAGAGCTAGAAATAGCAAG

Oligonucleotides used for genotyping

**Target**	**Forward (F)/Reverse (R)**	**Sequence (5' -> 3')**
**Genotyping of mutant plants**		
CAS9	F	CTTCGACCTGGCCGAAGATG
	R	CGTATTTGACCTTGGTGAGC
	F	CGGGAAGATGCGCTATCATG

Genomic RS2Z35	R	ATAGAACACAGCATGTGCAAGG
	R	CGACTAGTGTGTCTCATCTC
Genomic RS2Z36	F	ATGCCTCGTTATGATGATCGT
	R	TCACTGCAAATTCATCAGAGG
	R	AATGCTAGACCCTAAGGAACC
	F	AAGCACGACTATGCCTTCGT
**Genotyping of transgenic plants**		
GFP	F	ATGGGTAAAGGAGAAGAAC
	R	GGAACAGGTAGTTTTCCAGTAGTGCAAATAAA

Oligonucleotides used for RT-PCR and qRT-PCR

**Target**	**Forward (F)/Reverse (R)**	**Sequence (5' -> 3')**
**RT-PCR**		
HSFA2 (endogenous)	F	AGGCCGGATTCTGTTGTGAC
	R	GAGACCGCCTCAAAGCTTCCTG
HSFA2 (GFP-minigene)	F	GAGTTTGTAACAGCTGCTG
	R	GAGACCGCCTCAAAGCTTCCTG
**qRT-PCR**		
EF1α	F	TGATCAAGCCTGGTATGGTTGT
	R	CTGGGTCATCCTTGGAGTT
RS2Z35 (protein-coding)	F	GCGGTATGATGACAGGTATGG
	R	CCACATCACGTACTCTCCCA
RS2Z35 (endogenous)	F	TATCTCATCCTTGCACTTTGAT
	R	ATAGAACACAGCATGTGCAAGG
Oligonucleotides used for RT-PCR and qRT-PCR (continued)		
RS2Z36 (protein-coding)	F	AATAGCACTCGTCTCTATGTG
	R	GGATCACTAAATTCTACGAAGG
RS2Z36 (endogenous)	F	TGCAGTGCTTGGTATTTGATG
	R	TCACTGCAAATTCATCAGAGG
Hsp17.7A-CI	F	ATGGAGAGAAGCAGCGGTAA
	R	ATGTCAATGGCCTTCACCTC

HSFA2	F	TTCCACCACATTGTTGCCTA
	R	GCAAGCACCAGATCCTTGTT
